# Main Road Extraction from ZY-3 Grayscale Imagery Based on Directional Mathematical Morphology and VGI Prior Knowledge in Urban Areas

**DOI:** 10.1371/journal.pone.0138071

**Published:** 2015-09-23

**Authors:** Bo Liu, Huayi Wu, Yandong Wang, Wenming Liu

**Affiliations:** 1 State Key Laboratory of Information Engineering in Surveying, Mapping and Remote Sensing, Wuhan University, Wuhan city, PR, China; 2 Faculty of Geomatics, East China Institute of Technology, Nanchang city, PR, China; 3 Key laboratory of watershed ecology and geographical environment monitoring, National Administration of Surveying, Mapping and Geoinformation, East China Institute of Technology, Nanchang city, PR, China; Glasgow University, UNITED KINGDOM

## Abstract

Main road features extracted from remotely sensed imagery play an important role in many civilian and military applications, such as updating Geographic Information System (GIS) databases, urban structure analysis, spatial data matching and road navigation. Current methods for road feature extraction from high-resolution imagery are typically based on threshold value segmentation. It is difficult however, to completely separate road features from the background. We present a new method for extracting main roads from high-resolution grayscale imagery based on directional mathematical morphology and prior knowledge obtained from the Volunteered Geographic Information found in the OpenStreetMap. The two salient steps in this strategy are: (1) using directional mathematical morphology to enhance the contrast between roads and non-roads; (2) using OpenStreetMap roads as prior knowledge to segment the remotely sensed imagery. Experiments were conducted on two ZiYuan-3 images and one QuickBird high-resolution grayscale image to compare our proposed method to other commonly used techniques for road feature extraction. The results demonstrated the validity and better performance of the proposed method for urban main road feature extraction.

## Introduction

In recent years, earth observation technologies have led to a rapid development of satellite imagery at very high spatial resolution (such as ZiYuan-3, Geo-eye, QuickBird and World-view et al.). The ZiYuan-3 (ZY-3) satellite, launched in January 2012, is China’s first civilian high-resolution optical satellite and provides routine geospatial services for mapping, change monitoring, and resource surveying. The high spatial resolution and geometric accuracy of ZY-3 data products makes it easy to identify features such as roads and buildings in urban areas. Thus, these images have become an important data source for various geographic information applications. Accurate and up-to-date road information is essential for geographic information system (GIS) databases, transportation and urban planning, automated road navigation, and emergency response applications.

Rapidly changing urban environments such as those particularly evident in China precipitate the need for frequent updates and revisions of urban road databases. Many approaches have been developed to extract main roads from high resolution imagery in urban areas in order to keep the GIS road network databases updated in a timely way. Most of the research on road feature extraction from high resolution imagery has focused on aerial images, synthetic aperture radar (SAR) images and other satellite imagery [1–16]. Barzohar and Cooper [1] and Amo et al. [2] extracted roads from aerial images by using geometric-probabilistic models and estimation, and a region competition algorithm, respectively. Chanussot et al. [3] and Bentabet et al. [4] extracted roads from SAR satellite data to update vector road databases. Region-based road extraction methods have also been widely used [5–10]. Hu and Tao [5] automatically extracted highways from high resolution imagery by an energy minimizing based perceptual grouping method. Mokhtarzade and Zoej [8] developed a road detection method from high resolution satellite images using artificial neural networks. Huang and Zhang [9] extracted road centerlines from high-resolution images based on multi-scale structural features and Support Vector Machines (SVMs). To improve road feature extraction accuracy, other methods that use both spectral and spatial information have been proposed [11–16]. Song and Civco [11] used SVMs to first classify the imagery, and then detect road networks by shape features. Gamba et al. [12] and Grote et al. [13] proposed a road extraction method incorporating spatial information and road shape features. Miao et al. [14] and Shi et al. [15, 16] proposed urban road centerline extraction methods based on special-spatial classification and shape features. Although the integration of shape features is preferred in road extraction, it is difficult to achieve a shape feature extraction method suitable for all situations; therefore this is an active area of research [12].

Mathematical morphology (MM) is also one of the commonly used methods to extract roads from high resolution imagery [17, 18]. Zhu et al. [19] extracted road networks from high-resolution satellite images by using classical MM; however, their classical MM method depends on the shape of a Structuring Element (SE) and MM is not sufficient for detecting curved and rectilinear structures at the same time. In order to solve this problem, Chaudhuri et al. [20] used a customized structuring element to enhance the road structures in the image, and then used two road seed temples for segmentation. The results obtained from this enhancement of the MM segmentation approach therefore are significantly affected by the road seed template. Soille and Talbot [21] proposed directional morphological filtering to analyze oriented, thin, line-like objects. Heijmans et al. [22] proposed a flexible linear directional morphological filter—path openings and closings to detect oriented, thin, line-like objects in images. Talbot and Appleton [23] evaluated the effectiveness of path openings and path closings for detecting thin, elongated structures. Valero et al. [24] used path closings to detect road networks in very high resolution remote sensing images. In their method, a binary mask was used to extract the median gray value (*M*(*x*, *y*)) of roads in the image that was processed by path closings, and set *M*(*x*, *y*) as threshold to determine which pixels belonged to roads. *M*(*x*, *y*) was related to the location of the binary mask in the image. In a large image, a binary mask in different locations may get a different *M*(*x*, *y*), use a different *M*(*x*, *y*) to segment the image, and thus generate different segmentation results.

In this paper, we used directional mathematical morphology, Volunteered Geographic Information (VGI: [25]) captured in the OpenStreetMap (OSM: [26]) and shape features to more efficiently and accurately extract main roads in urban areas. VGI is a version of crowd-sourcing in which members of the general public create and contribute geo-referenced facts about the Earth's surface and near-surface to websites where these facts are synthesized into databases. Over the past few years, with the rise of the Web 2.0, VGI has undergone fast development [27]. One of the most well-known VGI projects is the OSM [26] that now provides abundant spatial, geometrical, and attribute information about road networks, especially those found in urban areas.

Our method integrates directional mathematical morphology, VGI prior knowledge captured in the OSM, and shape features to extract main road from ZY-3 images. The remainder of this paper is organized as follows. In section 2, our new method is presented. Experimental results are reported in section 3. Conclusions are drawn in section 4.

## Methodology

**Fig 1:**
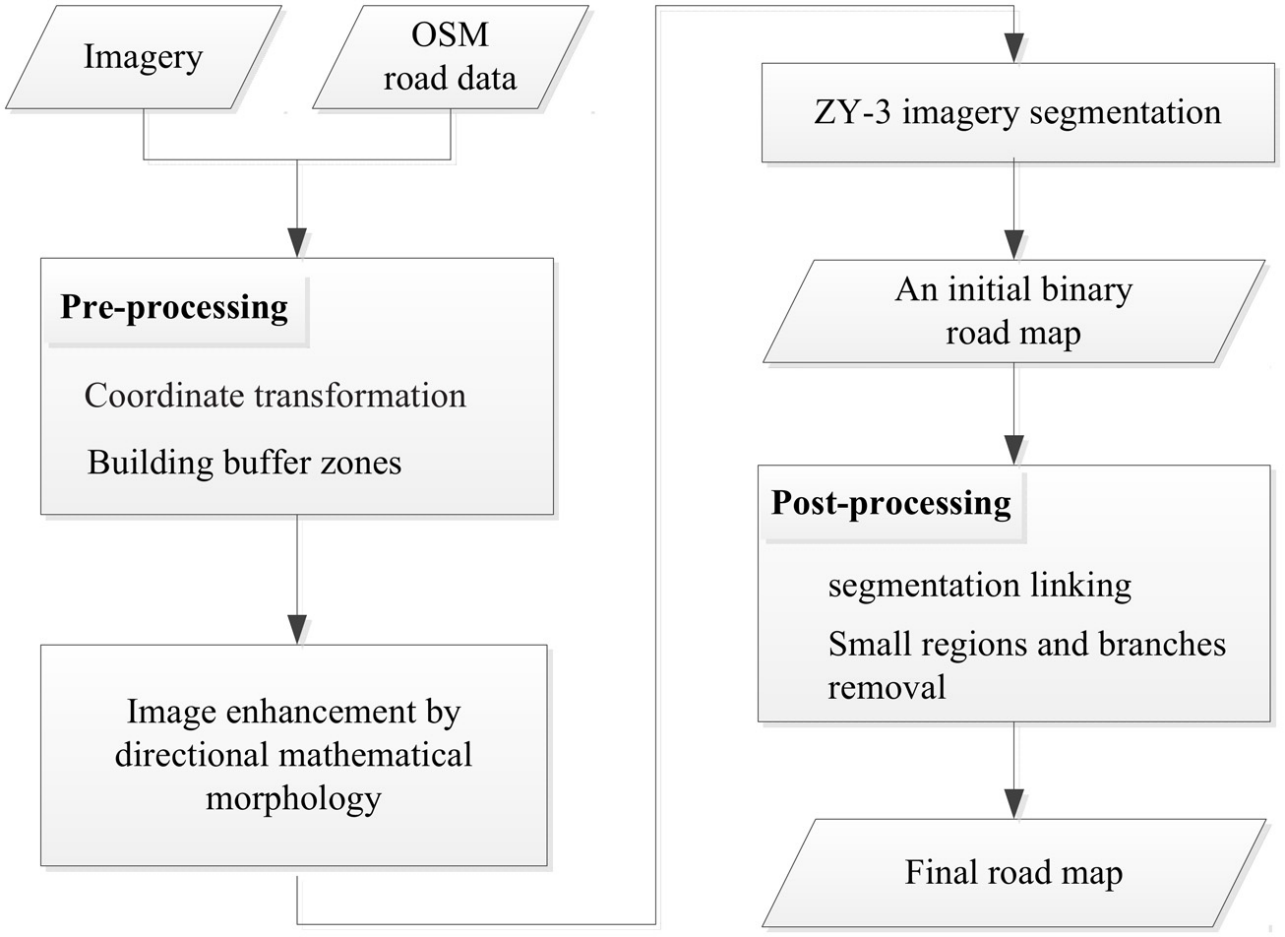
Flowchart of the proposed method.

A flowchart of our proposed method is shown in Fig 1. The details of each step are presented in detail in sub-sections 2.1 through 2.4.

As seen in Fig 1, there are four main stages in the proposed method. First, the pre-processing consists of two steps: coordinate transformation, and building buffer zones based on the OSM road network. Second, ZY-3 grayscale imagery is processed by path openings and path closings. Third, using the OSM road buffer zones as a region of interest (ROI), we create a ROI grayscale histogram. Then, the ZY-3 grayscale imagery is segmented based on the grayscale thresholds derived from the ROI grayscale histogram to generate an original binary road map. Post-processing consists of two steps: linking segments and small region and branch removal, to obtain a complete road network map.

### Pre-processing

Pre-processing consists of two steps: 1) Coordinate transformation. Generally, the coordinate system of OSM road map is World Geodetic System-84 (WGS-84) geographic coordinate system, and the projection is Web Transverse Mercator. The coordinate system of ZY-3 imagery is the China Geodetic Coordinate System 2000 (CGCS2000) and the projection is the universal transverse Mercator (UTM). In order to resolve the differences between the coordinate systems of OSM and ZY-3 imagery, we perform coordinate transformation, and convert WGS-84 to CGCS2000. 2) Build buffer zones. After coordinate transformation, we use the width attribution found in the OSM road network or a given empirical width value as buffer radius to build buffer zones. The buffer zones are overlaid on the ZY-3 imagery.

### Image enhancement by directional mathematical morphology

**Fig 2:**
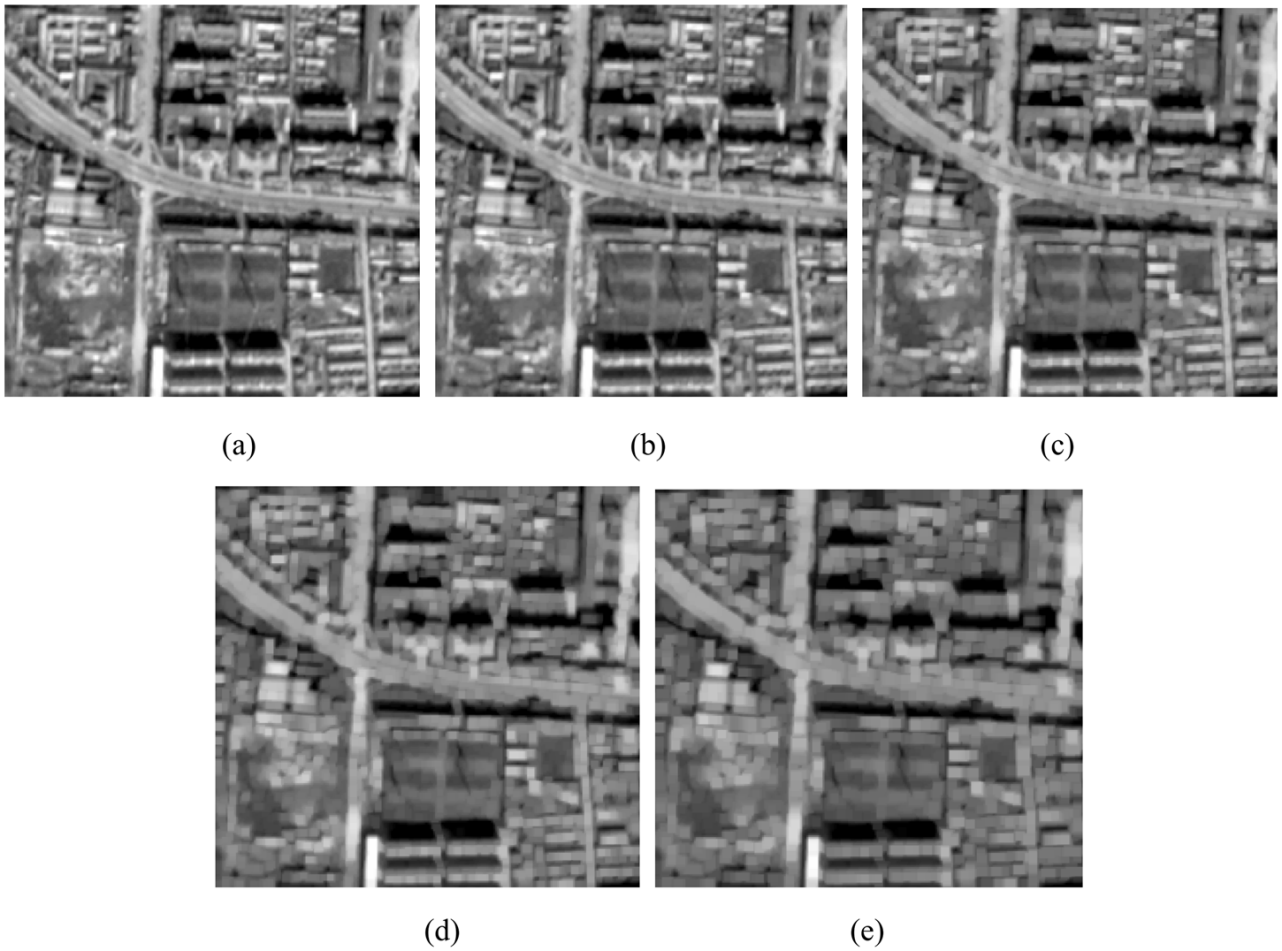
(a) Original image; (b) The result of the length of the SE *L* = 3; (c) The result of the length of the SE *L* = 5; (d) The result of the length of the SE *L* = 7; (e) The result of the length of the SE *L* = 9.

Image enhancement improves the contrast between the target and the background in high level processing including noise removal. In road extraction methodologies, mathematical morphological filtering is used to remove the noise and unwanted features, and preserve road segments as much as possible. However, classical mathematical morphological structuring elements are not flexible enough to detect rectilinear and curved structures at the same time [24]. Fig 2 shows image enhancement results as processed using the elongated shape of SEs. By increasing the length of the SEs, the objects (e.g., roads, buildings) get more blurred and distorted (e.g., the borders and the shapes). Nevertheless, most noise remains in the image, and it will still be difficult to extract road features from this image, despite image enhancement processing.

**Fig 3:**
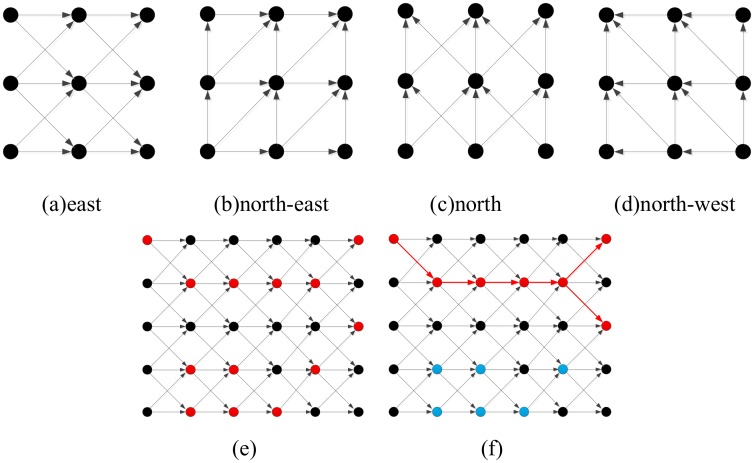
(a)-(d) Four different adjacencies; (e) Original data points are shown in red, and the adjacency is shown in gray; (f) The result of path openings with *L*_min_ = 5(red points at the right), the discarded points are shown in blue.

To remedy this limitation, Chaudhuri et al. [20] proposed a customized 5×5 structuring element that considered four directions (horizontal, right diagonal, left diagonal and vertical) to enhance the road structures in the image. But, if the width of the road is less than five pixels, the proposed enhancement method cannot handle it well. Soille and Talbot [21] proposed directional morphological filtering to analyze oriented, thin, line-like objects, while a flexible directional linear morphological filter——path openings and path closings was proposed by Heijmans et al. [22], to address this problem. Path openings and closings replace classical MM SEs with graph connectivity as shown in Fig 3. Fig 3a–3d show four different adjacency relationships, while Fig 3e and 3f present an example of path openings.

In general, roads are in the shape of a long narrow rectangle or a band-shaped line, and appear as linear features in the image. The characteristics of roads are very suitable for using path openings and path closings, so the purpose of this step is to enhance the contrast between the road and non-road features by using path openings and path closings. Detailed information about path openings and path closings is presented in related references [22, 23].

**Fig 4:**
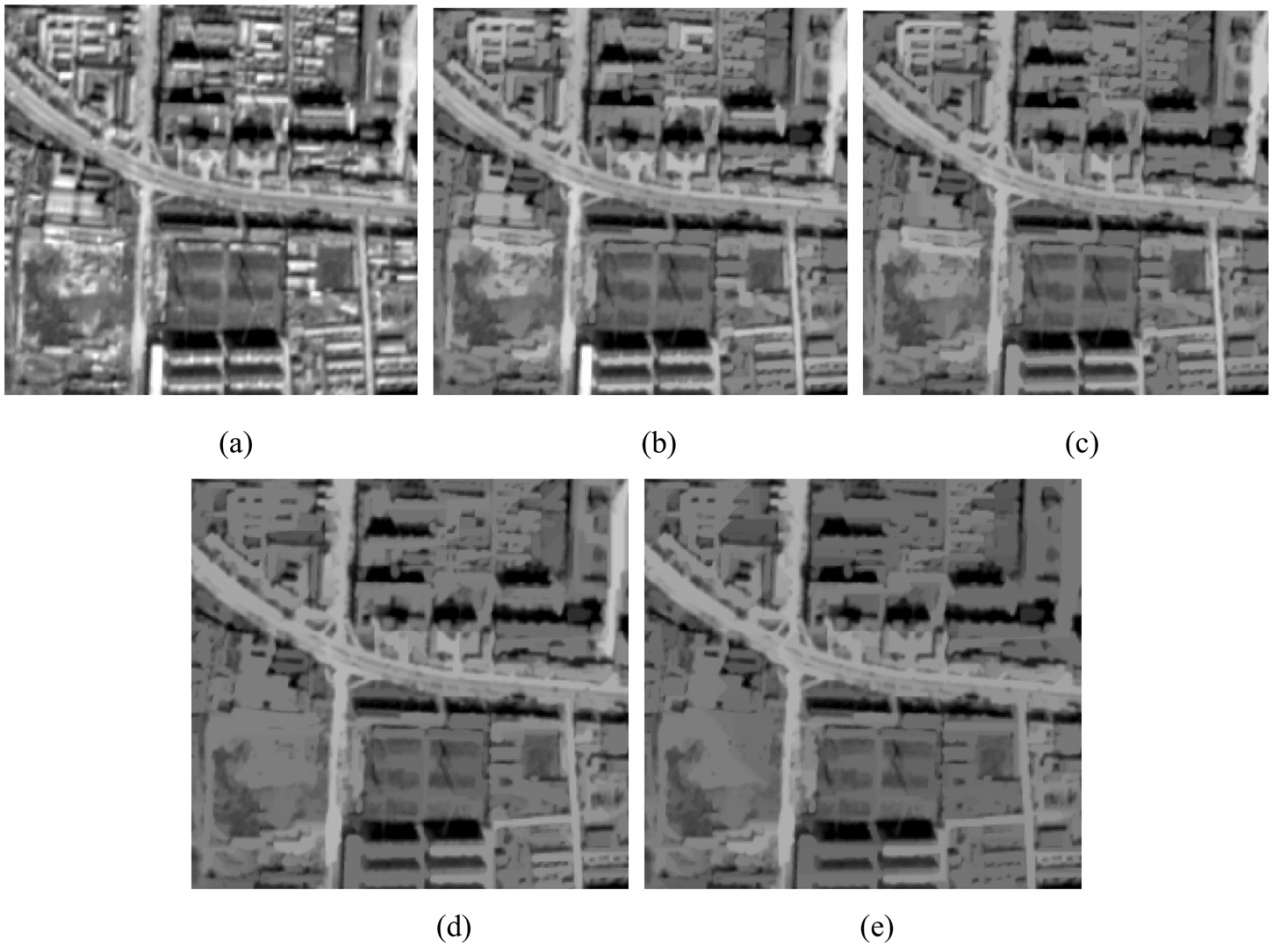
(a) Original image; (b) The result of path opening with *L*_min_ = 50; (c) The result of path opening with *L*_min_ = 100; (d) The result of path opening with *L*_min_ = 200; (e) The result of path opening with *L*_min_ = 300.

Fig 4 shows the results from Fig 2a as processed by path openings with different path lengths. In this example, much of the noise in the image has disappeared and the contrast between road and non-road features has been enhanced through an increase in path length.

We instead use path openings and path closings to enhance the image. The specific steps consist of processing path openings followed by path closings to remove non-road noise. Because a morphological filter consisting of opening followed by closing can eliminate noise that manifests as randomly placed bright elements in a dark background and or as dark elements in a bright background [20]. After enhancement, we can now use a threshold to segment the image to obtain an initial binary road map.

### ZY-3 imagery segmentation

For grayscale images, threshold segmentation is a simple but effective tool to separate objects from the background. Many image segmentation methods have been discussed and proposed for various applications, but clustering threshold segmentation methods are better for grayscale image segmentation [28]. Otsu [29] is one of the most popular clustering threshold segmentation methods; so far, this method still remains one of the most referenced thresholding methods [28]. Otsu suggested minimizing the weighted sum of within-class variances of the foreground and background pixels to establish an optimum threshold; minimization of within class variances is tantamount to the maximization of between-class scatter. This method gives satisfactory results when the numbers of pixels in each class are close to each other. Although pre-processing eliminates much of the non-road noise, it is still difficult to obtain the main road from the ZY-3 grayscale imagery.

**Fig 5:**
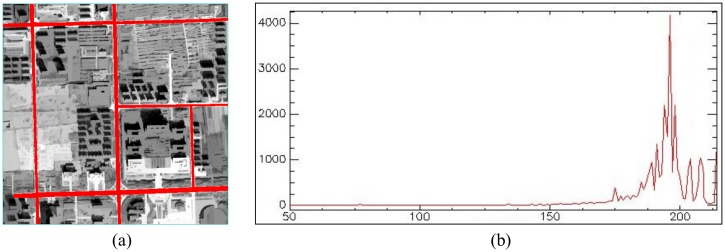
(a) OSM road buffer as ROI overlay on the image; (b) grayscale histogram of ROI.

In this section, we use a new segmentation technique that overcomes the limitations of the existing methods to segment the ZY-3 grayscale imagery, and transform it to an initial binary road map for further processing and road feature extraction. We use OSM roads as prior knowledge to obtain the gray value of roads, and use the gray value as the threshold to segment the ZY-3 grayscale imagery. The main steps include: 1) Overlay the OSM road buffer zones obtained in the pre-processing section on the ZY-3 grayscale image processed by the path openings and path closings; 2) Use the buffer zones as ROI, and obtain the grayscale histogram of ROI in the imagery; 3) Find the min value(*x*_min_), max value(*x*_max_), mean value($\bar{x}$) and standard deviation value (*δ*)of the gray value in the histogram, and set the gray value *x* as the threshold for the imagery segmentation, thus *x* is $\bar{x}-\delta \leq x\leq \bar{x}+\delta$ or $x\in [\bar{x}-\delta ,\bar{x}+\delta ]$. Subsequently, when the gray value is between $\bar{x}-\delta$ and $\bar{x}+\delta$, pixels can be seen as road features, other pixels can be seen as non-road features. For instance, Fig 5a shows the OSM road buffer as an ROI overlaid on the image, while Fig 5b shows the grayscale histogram of ROI. The min value (*x*_min_), max value (*x*_max_), mean value ($\bar{x}$), and standard deviation value (*δ*) of the gray value in the histogram are shown in Table 1. In Fig 5a, the threshold *x* for the imagery segmentation is *x* ∈ [176,210]; therefore, when the gray value of one pixel is between 176 and 210 in the image, the pixel is considered to be a road, and pixels outside that range are treated as non-road features.

**Table 1:** Basic statistics from the grayscale histogram.

Basic statistics	Min(*x*_min_)	Max(*x*_max_)	Mean($\bar{x}$)	StDev(*δ*)
Band 1	50	214	193	17

Using the OSM in automatic generation of a threshold value is a feasible way to incorporate prior knowledge into image segmentation. As timely volunteered geographic information, the OSM embodies a sort of ground truth and might be, in many instances, the only information available about roads in certain urban areas. Therefore, using OSM data to construct the threshold makes it independent from the location in the image.

### Post-processing

There are many small area regions, and short and discontinuous road segments, in the initial binary road map created during processing as described in section 2.3. A road whose width is greater than 4m, and length is greater than 50m is considered to be a “main road” feature. Small area regions, however, as well as short or narrow road segments, are considered to be “non-main-road” features, even though in fact they are parts of roads in the road network. Main roads can be in the shape of a long narrow rectangle or as band-shaped lines. A rectangle or band-shaped lines have orientations, lengths, and widths. Usually, the width of a road is several pixels in high-resolution satellite images. The length of a road is usually longer than buildings and longer than or equal to a street block. In this section, post-processing is used to merge these discontinuous road segments, and to remove remaining non-main-roads. As shown in Fig 1, the steps of post-processing include: 1) segmentation linking; 2) small region and branch removal.

#### (1) Road segments linking

There may be discontinuous road segments in the initial road map. We use a linking procedure to connect them. Our linking procedure depends on the relative orientation and distance of road segmentations.

**Fig 6:**
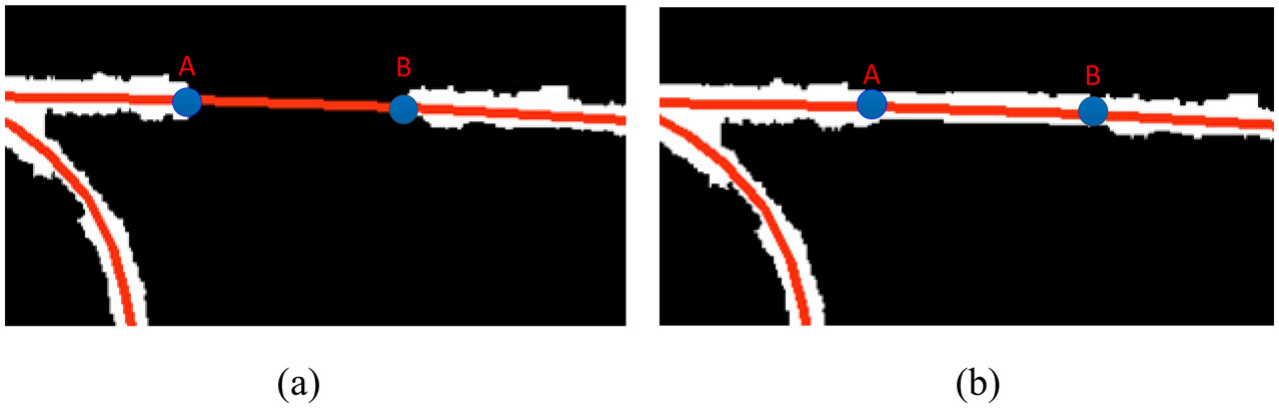
(a) two disconnected road segments (red line segment is OSM road); (b) linking result.

There are two cases when linking road segments: 1) OSM roads act as prior knowledge that provides the orientation, length, and width of discontinuous road segments; we can use this prior knowledge to merge discontinuous road segments. As shown in Fig 6a, A and B are two end pixels of the disconnected road segment, while A, B are also two nodes of OSM road networks (the red line segment in Fig 6 is an OSM road). According to the geometrical information from line segment AB, we calculate the distance (*D*_*AB*_) between A and B, and the orientation (*O*_*AB*_) of line AB. Along the direction of AB, we locate the pixels that need to be mapped as road pixels; the results processed by the linking procedure are shown in Fig 6b. Compared with the linking method proposed by Chaudhuri et al. [20], in our research, if we have OSM roads as prior knowledge, we do not need to set the threshold of the distance and angle. 2) Whereas, if there is no prior knowledge, we use the linking method as proposed by Chaudhuri et al. [20] to connect discontinuous road segments. The details of this link method can be found in [20].

#### (2) Remove small regions and branches

There are many small area non-road regions and short length road segments associated with the main roads in the road map. The small non-road linear structures are associated with the main road skeleton but must be removed. The lengths of these structures are significantly smaller than the length of the main road. Connected component analysis (CCA) was used for small branch removal [9]. CCA groups pixels into connected components based on pixel connectivity (8-neighbours) and calculates the geometrical attributes for each component such as area, perimeter, Euler number, and orientation. We also use the shape parameters of roads to remove regions with a small area and branches, introducing these road shape features by: 1) area; 2) compactness; and 3) elongation.

1) Area: A road is commonly a continuous feature with a relatively larger area than other man-made features. Hence, segments with small area values can be viewed as a non-road class and removed.

2) Compactness: A road is in the shape of a long narrow rectangle or a band-shaped line; the width of a road is several pixels in high-resolution satellite images. Hence, compactness is expressed by the compactness index (*CI*) as follows:

$$CI=\sqrt{\frac{4*S_{i}}{\pi }}/L_{i}$$ (1)

Where *S*_*i*_ is the area of an object and *L*_*i*_ represents the outer contour length of an object. A circle is the most compact shape with a value of 1/*π*. The *CI* of a square is $1/2\sqrt{\pi }$. Road segments have small values of *CI*.

3) Elongation: Similar to compactness, the elongation index (*EI*) is defined as follows:

$$EI=L_{major}/L_{\text{min}or}$$ (2)

Where *L*_*major*_ is the length of the major axis of an object and *L*_minor_ is the length of the minor axis of an object. The major and minor axes are derived from an oriented bounding box containing the object. The EI for a square is 1.0, and the EI for a rectangle is greater than 1.0. Thus, road segments have large values of *EI*, because of the large length of the major axis and small length of the minor axis.

In our research, the shape parameters for each component were computed, and each component that satisfied Eq (3) was judged as road, the others were deleted as non-road features.

$$S_{Area}\geq T_{Area}\text{ and }S_{CI}\leq T_{CI}\text{ and }S_{EI}\geq T_{EI}$$ (3)

Where *S*_*Area*_, *S*_*CI*_ and *S*_*EI*_ denote the *Area*, *CI*, and *EI* values of the segments, respectively. *T*_*Area*_, *T*_*CI*_, and *T*_*EI*_ are the corresponding thresholds that are empirically determined. In the end, we also used the standard mathematical morphology method to fill holes in the roads.

## Experiments and Analysis

In order to evaluate the proposed method, we conducted several tests. Two experiments were conducted to extract main roads from the ZY-3 grayscale satellite imagery. The spatial resolution was 2 m per pixel. Another experiment was conducted to extract main roads from a QuickBird grayscale image. The spatial resolution was 0.6m per pixel.

### Experiment I

**Fig 7:**
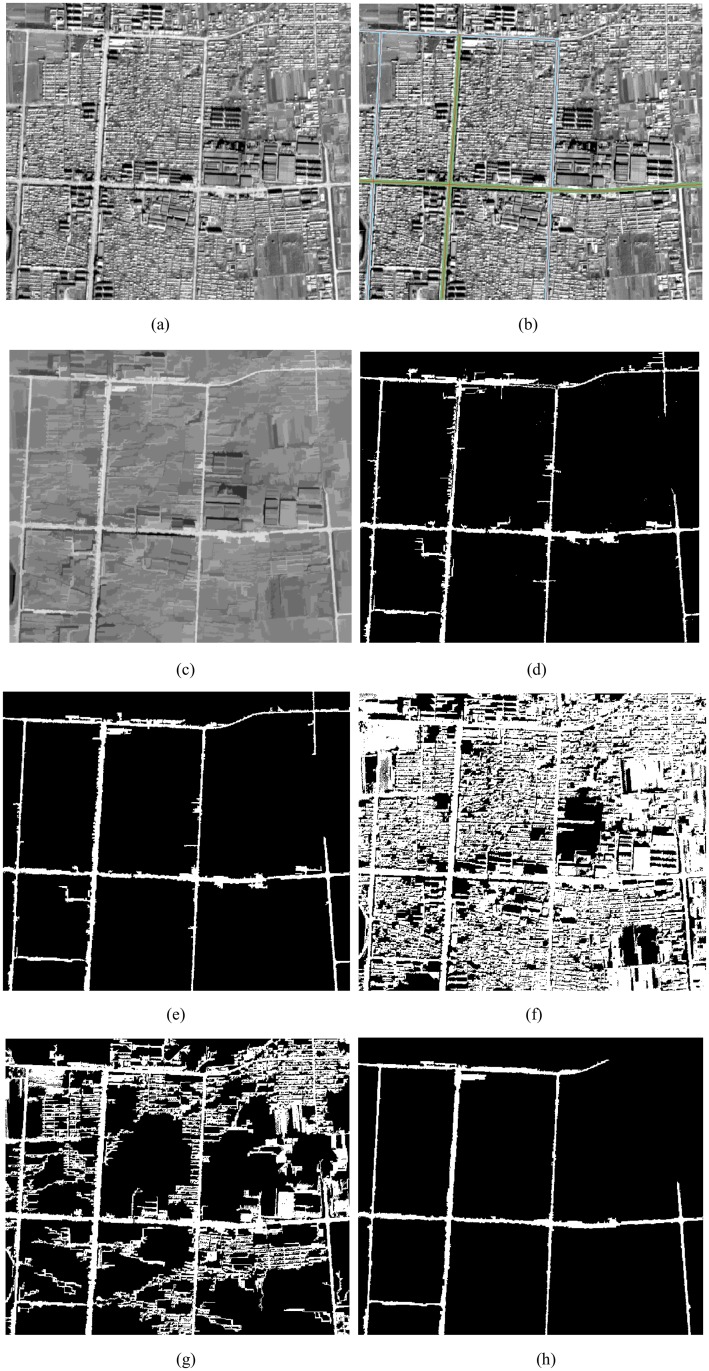
(a) Test imagery; (b) OSM road buffer zones as ROI overlay the original test imagery; (c) image processed by image enhancement with *L*_min_ = 100; (d) image segmentation, the threshold value *x* ∈ [178,226]; (e) the result after post-processing; (f) the result processed by Otsu method for the enhanced image; (g) the result processed by Valero et al. [24]; (h) the result processed by Chaudhuri et al. [20].

The original input ZY-3 grayscale imagery was an 8-bit grayscale 617*531 (i.e., 256 luminosity levels) image as shown in Fig 7a, an old district of YingKou city. There are many buildings in the image. In the test image, besides the main roads, there are some narrow roads located in the upper right corner. The gray values of some buildings are similar to the main roads.

As discussed in section 2.1, we first unify the coordinate system between the OSM roads and test image, and set the OSM road width value as the radius to build the buffer. At the same time, we set the OSM roads and the buffer overlay on the test image as shown in Fig 7b. In Fig 7b, the red lines are OSM roads with different width values. The green and blue polygons are the buffer zones for the OSM roads; the radius of each buffer zone is consistent with the width of the road. Second, we overlay the buffer zones on the image processed by image enhancement with *L*_min_ = 100 as shown in Fig 7c. We use the buffer zones as the region of interest (ROI) to obtain a grayscale histogram. In the histogram, the *X*-axis represents the luminosity level; the *Y*-axis has histogram values that represent the number of pixels for each luminosity level. The min value (*x*_min_), max value (*x*_max_), mean value ($\bar{x}$) and standard deviation value (*δ*) of the gray are 1, 236, 202 and 24 respectively. We set the gray value *x* ∈ [178,226] as road features, while other gray values are defined as non-road features; the results are shown in Fig 7d. From Fig 7d, it can be seen that most noise such as roofs, shadows, and bare soil have been removed, but there are still a few small branches and some regions with small areas. Further processing is needed to remove this noise. The results after post-processing are shown in Fig 7e; the values of *T*_*Area*_, *T*_*CI*_, and *T*_*EI*_ are 30, 0.15 and 2.0 respectively. Fig 7f shows the image segmentation results using the technique proposed by Otsu [29] on the enhanced image. Fig 7g shows the road extraction result by the method proposed by Valero et al. [24]. Fig 7h shows the road extraction results by the method proposed by Chaudhuri et al. [20].

### Experiment II

**Fig 8:**
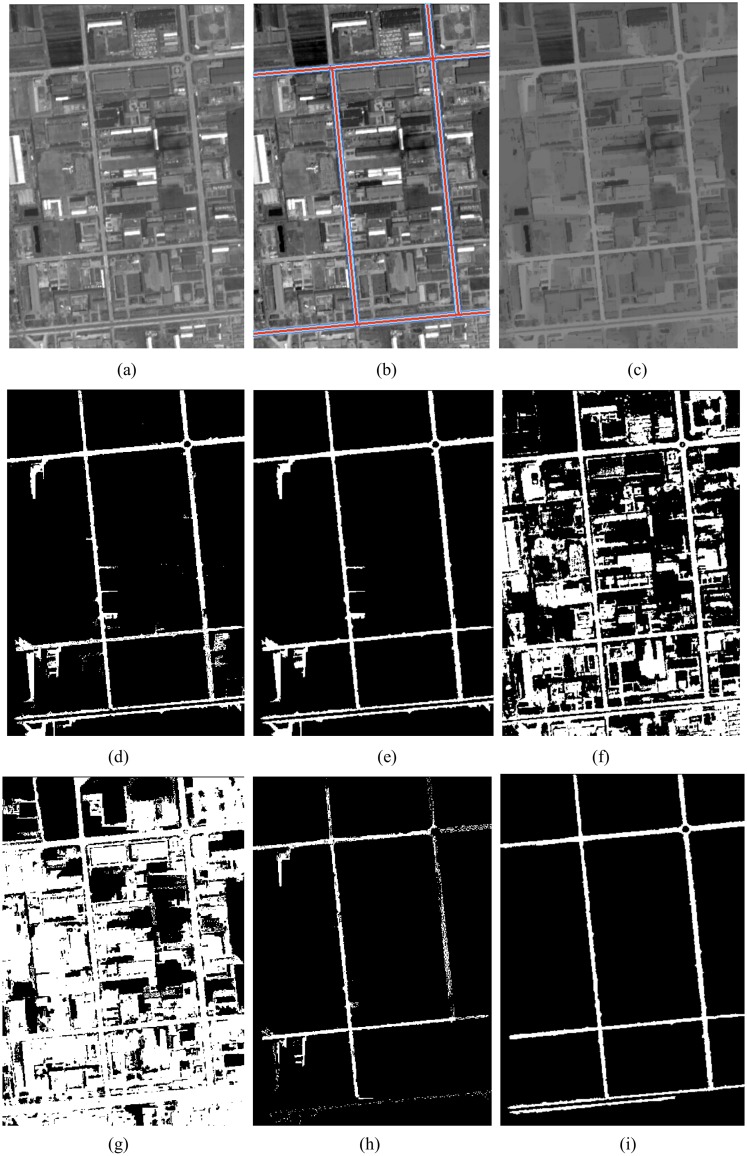
(a) Test imagery; (b) OSM road buffers as ROI overlay the original test imagery; (c) image processed by image enhancement with *L*_min_ = 300; (d) image segmentation, the threshold *x* ∈ [132,148]; (e) the result after post-processing; (f) the result processed by Otsu method for original test imagery; (g) the result processed by Otsu method for enhanced image; (h) the result processed by Valero et al. [24]; (i) the result processed by Chaudhuri et al. [20].

The test image is an 8-bit ZY-3 grayscale 344*501 (i.e., 256 luminosity levels) image as shown in Fig 8a, a new industrial district of JiNan city. In the test image, the width of the main roads is consistent, but there are many big factory buildings in the imagery that look like roads.

As in the previous experiment, we create the buffer zones according to the width of the OSM road, and overlaid the buffers on the original test image as shown in Fig 8b. Fig 8c shows the results processed by image enhancement with *L*_min_ = 300. The image segment results are shown in Fig 8d. As in experiment I, we use the buffer zones as the ROI to obtain the road gray histogram. In the histogram, the min value (*x*_min_), max value (*x*_max_), mean value ($\bar{x}$) and standard deviation value (*δ*) of the gray are 80, 163, 140 and 8 respectively. We set the gray value to *x* ∈ [132,148] as the imagery segmentation threshold value. The results after post-processing are shown in Fig 8e. The values for *T*_*Area*_, *T*_*CI*_, and *T*_*EI*_ are 50, 0.15 and 2.5 respectively. Fig 8f shows the image segmentation results obtained by using the technique proposed by Otsu [29] on the original image. Fig 8g shows these image segmentation results on the enhanced image. Fig 8h shows the road extraction result by the method proposed by Valero et al. [24]. Fig 8i shows the road extraction results by using the method proposed by Chaudhuri et al. [20].

### Experiment III

**Fig 9:**
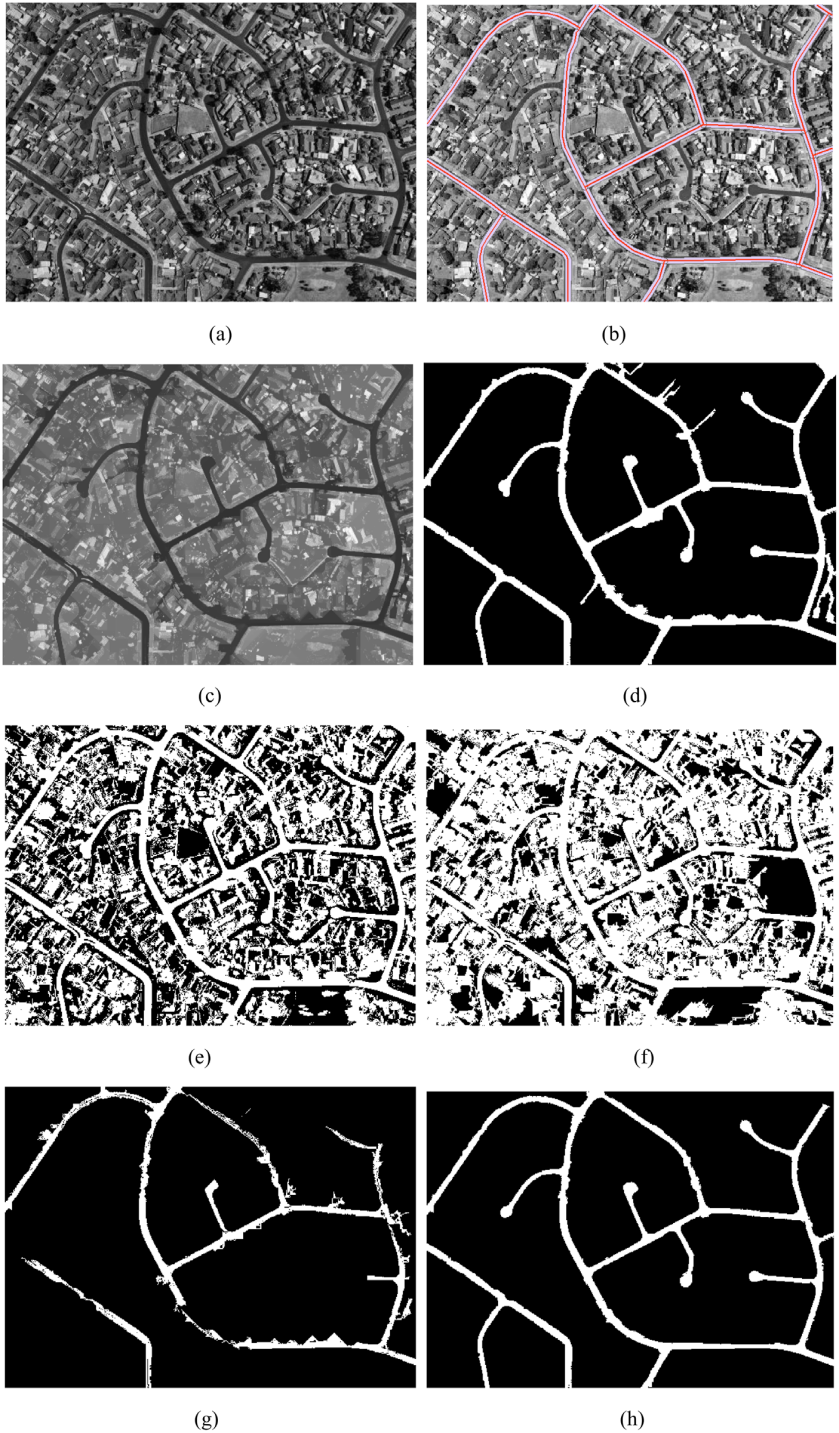
(a) Test imagery; (b) OSM road buffers as ROI overlay the original test imagery; (c) image processed by image enhancement with *L*_min_ = 200; (d) the result processed by the proposed method; (e) the result processed by Otsu method for original test imagery; (f) the result processed by Otsu method for path openings imagery; (g) the result processed by Valero et al. [24]; (h) the result processed by Chaudhuri et al. [20].

The test image is an 8-bit QuickBird grayscale 537*394 (i.e., 256 luminosity levels) image (the spatial resolution was 0.6m per pixel.) as shown in Fig 9a, It depicts a residential area of Sydney, Australia. As in the previous experiments, we create the buffer zones according to the width of the OSM roads, and overlay the buffers on the original test image as shown in Fig 9b. Fig 9c shows the results processed by image enhancement with *L*_min_ = 200. As experiment I, we use the buffer zones as the ROI to obtain the road gray histogram. In the histogram, the min value (*x*_min_), max value (*x*_max_), mean value ($\bar{x}$) and standard deviation value (*δ*) of the gray are 36, 253, 60 and 12 respectively. We set the gray value to *x* ∈ [48,72] as the imagery segmentation threshold value. The results after post-processing are shown in Fig 9d. The values for *T*_*Area*_, *T*_*CI*_, and *T*_*EI*_ were 60, 0.15, and 2.5 respectively. Fig 9e shows the image segmentation results obtained by using the technique proposed by Otsu [29] on the original image. Fig 9f shows the image segmentation results obtained by using the technique proposed by Otsu [29] on the enhanced image. Fig 9g shows the road extraction results using the method proposed by Valero et al. [24]. Fig 9h shows the road extraction results from the method proposed by Chaudhuri et al. [20].

### Analysis

Several observations can be made on the basis of comparison of the performance of the proposed method and existing methods. In experiment I, II and III, the results from threshold segmentation for the original test imagery processed by the Otsu method, without any post-processing, are shown in Figs 8f and 9e, respectively. The results of threshold segmentation for enhanced image processed by the Otsu method on the two ZY3 images and one QuickBird image without any post-processing are shown in Figs 7f, 8g and 9f respectively. Although, Otsu [29] proposed one of the most popular clustering threshold segmentation methods, it is designed for image segmentation purposes, not for road extraction from satellite images. From these figures, it can be seen that the Otsu method can identify some main roads, but there is still a great deal of difficulty to remove non-road noise in the image.

Figs 7g, 8h and 9g show the results of the method proposed by Valero et al. [23] on the two ZY3 images and one QuickBird image. From these figures, it can be seen that the Valero et al. [23] method, which uses the median gray value from the binary mask as threshold to segment the image, can identify main roads, but these results are not complete or visually satisfying. In Fig 7g, much non-road noise appears in the image despite processing. In Figs 8h and 9g, where only the median gray values were used to segment the image, many roads cannot be detected because the gray values of all roads were not consistent. In order to improve this shortcoming, we considered the following characteristics of roads: 1) after processing by path openings and path closings, the gray values change smoothly inside the road, and a gray histogram of the road usually has one main peak. In general, the distribution of the gray values in the histogram follows a normal distribution; 2) the difference of the gray values between the roads and background is bigger in the image when processed by path openings and path closings; and 3) the difference of gray values between roads and background displayed on the gradient histogram has non-zero peaks. In our method, based on the gray histogram of road, we set $\left[\bar{x}-\delta ,\bar{x}+\delta \right]$ as threshold to segment the image; the results without post-processing are shown in Figs 7d, 8d and 9d, respectively. Most of the main roads were detected by the proposed method and the images produced by our proposed method were more complete and visually superior to those produced by the method proposed by Valero et al. [24].

Figs 7h, 8i and 9h show the results of the method proposed by Chaudhuri, D. et al. [20] on two ZY-3 images and one QuickBird image. From Figs 7h, 8i and 9h, it can be seen that the main road was detected, and the images demonstrate that the method is highly accurate. Especially, the results as shown in Fig 9h, detected from QuickBird 0.6m imagery, are more accurate than the results as shown in Fig 9d. By comparing the results that are shown in Fig 7d and 7h, Fig 8d and 8i, it can be seen that some thin roads (less than five pixels wide) are not detected as Chaudhuri et al. [20] used a customized 5×5 directional structuring element as road seed template to enhance image, and then used two 5×5 road seed templates to segment the image. For the ZY-3 images used in our experiments, the spatial resolution is 2m, thus a road would not be detected if the width was less than 10m. So, in Figs 7h and 8i, some roads are not detected. For the QuickBird image used in our experiments, the spatial resolution is 0.6m, and the width of road is bigger than 5 pixels, so the roads detected result from the method proposed by Chaudhuri et al. [20] is more accurate than the result processed by our method.

At the same time, from Figs 7e, 8e and 9d, it can be seen that our proposed method detected many non-road features (e.g. buildings and shade) as road features. In contrast to the image enhancement technology proposed by Chaudhuri et al. [20], in our proposed method, there is no need to choose a 5×5 road template as structuring element. Because path openings and path closings are related to the length of the “path” (*L*_min_), the enhancement technology also enhanced all the elongated objects that with length greater than *L*_min_, and the segmentation technology just used the grayscale threshold to detect whether each pixel belonged to a road or not. This also created some non-road noise.

The directional mathematical morphology (path openings and path closings) depended on the length of the path (*L*_min_) to remove the non-road noise. In experiment I, the road structures were bright, and the test image had many small buildings and small roads connected to the main roads, therefore, we set the length of the path as *L*_min_ = 100. After processing by path openings and path closings, most small buildings, small roads, and other non-road noise were dimmed as shown in Fig 7c. The contrast of the main road and the other objects surrounding the main road was very strong. In experiment II, the road structures were bright, the test image had many big factory buildings, and the main roads were long and wide. We therefore set the length of the path as *L*_min_ = 300. After processing by path openings and path closings, most factory buildings and other non-road noise were dimmed as shown in Fig 8c; the contrast of the main road and the other objects surrounding the main road was strong. In experiment III, the road structures were dark, and the test image had many buildings, and the main roads were long and wide. We therefore set the length of the path as *L*_min_ = 200. After processing by path openings and path closings, most factory buildings and other non-road noises were dimmed as shown in Fig 9c, as the contrast of the main road and the other objects surrounding the main road was strong.

Given the characteristics of roads, we used OSM roads as prior knowledge to obtain the gray values of roads in the test images, and then used these values as the threshold value to segment the test images, at the same time; we also used the orientation, length and width of roads obtained from OSM roads to link discontinuous road segments, the results were shown in Figs 7e, 8e and 9d, respectively. From these experimental results it could be seen that using OSM roads as prior knowledge helps us detect the main roads and in our results, most non-road noise has been removed.

Although VGI has undergone fast-paced development worldwide, the quality and update speed of VGI is still one of the important problems. Figs 7b, 8b and 9b illustrate that the OSM roads match only parts of the main roads, so taking advantage of remote sensing image data to supplement and update OSM roads is very necessary. As shown in Figs 7e, 8e and 9d, the proposed method can get most main roads, and can solve the quality and update speed problems found in VGI, further promoting the development of this rich data source.

## Conclusions

In our research, a main road extraction method for grayscale imagery was presented. The proposed method uses directional mathematical morphology and VGI prior knowledge to extract main roads. The main steps in our algorithm are: coordinate transformation, building buffer zones, path openings and path closings, imagery segmentation, and small regions and branches removal. The proposed algorithm was evaluated by using ZY-3 and QuickBird grayscale images; the results demonstrate that the algorithm is highly accurate. The proposed method, based on the directional mathematical morphology and OSM roads as prior knowledge, is feasible and effective for urban main road extraction, and many man-made structures having a spectral reflectance similar to that of roads can be satisfactorily eliminated using threshold segmentation. As compared with other methods, the difficulty of post-processing was greatly reduced, and using VGI as prior knowledge helped us obtain the threshold to segment enhanced images, the results were better than the other methods we tested.

Since the proposed method is based on a threshold to segment the image, it is suitable for high-resolution imagery, and when the roads have the same structures (e.g., the road structures are dark or bright), but it is not suitable for low-resolution imagery, or when the roads have different road structures (e.g., the road structures are dark and bright). Another limitation of the proposed method is that the length of the path (*L*_min_), *T*_*Area*_, *T*_*CI*_ and *T*_*EI*_ have to be determined manually. An objective and automatic threshold-determination method and more optimal segmentation technology will be studied in future research.
